# Developing and demonstrating an atomistic and holistic model of anthropometric failure among children under five years of age using the National Family Health Survey (NFHS)-5 data from India

**DOI:** 10.3389/fnut.2023.1280219

**Published:** 2024-01-08

**Authors:** E. R. Nandeep, Abdul Jaleel, P. Bhaskar Reddy, J. J. Babu Geddam, Samarasimha N. Reddy, Rajkumar Hemalatha

**Affiliations:** ^1^Clinical Epidemiology Division, Indian Council of Medical Research (ICMR)-National Institute of Nutrition, Hyderabad, Telangana, India; ^2^Public Health Nutrition Division, Indian Council of Medical Research (ICMR)-National Institute of Nutrition, Hyderabad, Telangana, India; ^3^Nutrition Information, Communication & Health Education (NICHE) Division, Indian Council of Medical Research (ICMR)-National Institute of Nutrition, Hyderabad, Telangana, India; ^4^Indian Council of Medical Research (ICMR)-National Institute of Nutrition, Hyderabad, Telangana, India

**Keywords:** malnutrition, undernutrition, Z-score, National Family Health Survey-5, under-five children, anthropometric failure

## Abstract

**Introduction:**

Composite Index of Anthropometric Failure (CIAF) and its further modifications have not incorporated all the combinations of malnutrition. We propose a new model incorporating all the forms of malnutrition among children under five years of age. However, the current models might misclassify a growing child as malnourished. Our objective is to develop a comprehensive scoring system using the three anthropometric Z-scores [height-for-age (HAZ), weight-for-age (WAZ), and weight-for-height (WHZ) Z-scores] and demonstrate the proposed CIAF model using the National Family Health Survey-5 (NFHS-5) data from India.

**Methods:**

A new scoring system was developed using the WAZ, HAZ, and WHZ scores to determine the child’s nutritional status. We also proposed a new CIAF model by including all possible categories of malnutrition and practically demonstrated it using the NFHS-5 dataset after applying the new scoring system. Under-five children with heights, weights, and ages available were included in the analysis. The groups of malnutrition are presented as weighted proportions before and after applying the new score to the proposed model.

**Results:**

Our final analysis included individual-level data of 198,802 children under five years of age (weighted *N* = 195,197). After applying the new scoring system to the proposed model, the prevalence of stunting has reduced to 11.8% (95% CI 11.66–11.94) from 13.2% (95% CI 13.09–13.39) and wasting prevalence has reduced to 4.9% (95% CI 4.85–5.04) from 6.4% (95% CI 6.29–6.51). The most common forms of anthropometric failures among Indian children by using the newly developed CIAF model are: “Stunting and underweight” (30,127; 15.4%), Stunting only (23,035; 11.8%), and “wasting and underweight” (14,698; 7.5%). We found a new category called “Stunting, underweight, and overweight” (stunting = HAZ < −2SD, underweight = WAZ < −2SD, overweight = WHZ > +2SD). It constituted 0.1% (220 children) of the total sample.

**Conclusion:**

When the new scoring system is applied to the proposed CIAF model, it captures all forms and combinations of malnutrition among under-five children without overlap and prevents misclassifying a growing child as malnourished. The newly identified category shows that stunting (HAZ < −2SD), overweight (WHZ > +2SD) and underweight (WAZ < −2SD) can co-exist in the same child.

## Introduction

The use of standard deviation (SD) and Z-scores are a good method to reflect nutritional status among children with the development of the World Health Organization (WHO) growth charts (1). Each of stunting [< −2SD height-for-age Z-score (HAZ)], wasting [< −2SD weight-for-height Z-score (WHZ)], underweight [< −2SD weight-for-age Z-score (WAZ)], and overweight (WHZ > +2SD) provides useful information about a different biological entity (2).

**FIGURE 1:**
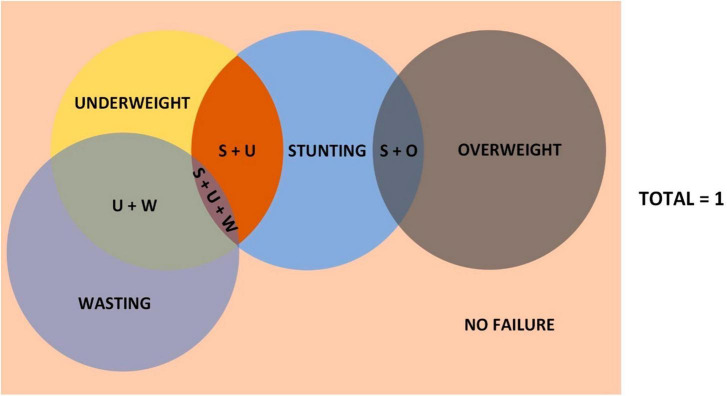
Kuiti’s model of CIAF in Venn diagram. Not to scale. U + W, underweight and wasting; S + O, stunting and overweight; S + U, stunting and underweight; S + U + W, stunting, underweight, and wasting.

None of the conventional indicators captures the sum of all the children who have anthropometric failure in at least one of the measures considered (height-for-age, weight-for-height, weight-for-age) (3). The “Composite Index of Anthropometric Failure” (CIAF) was proposed by Peter Svedberg to address this issue. In simple terms, CIAF is one minus the proportion of children with no failure of any form (3). An additional Group Y which is “Underweight only” was recognized by Nandy et al. (4) and added to Svedberg’s model while operationalizing CIAF for the first time (We would henceforth be referring to “Svedberg’s model” as this new model after the addition of “Underweight only” category, and not the initial model Svedberg suggested) (Table 1). However, this model of CIAF does not address the issues of overweight and obesity, and hence was not sufficient to find out the overall burden of malnutrition. Hence, Kuiti and Bose et al. (5) proposed two additional groups to the then-existing model: Stunting with Overweight; and Overweight only (Group G and H, respectively, in Table 2) (Figure 1).

**TABLE 1:** Groups in Svedberg’s model of CIAF.

Group	Wasting	Stunting	Underweight
A: No failure	No	No	No
B: Wasting only	Yes	No	No
C: Wasting and underweight	Yes	No	Yes
D: Wasting, stunting, and underweight	Yes	Yes	Yes
E: Stunting and underweight	No	Yes	Yes
F: Stunting only	No	Yes	No
Y: Underweight only	No	No	Yes

**TABLE 2:** Kuiti model of CIAF with two new groups G and H.

Group	Wasting	Stunting	Underweight	Overweight
A: No failure	No	No	No	No
B: Wasting only	Yes	No	No	No
C: Wasting and underweight	Yes	No	Yes	No
D: Wasting, stunting, and underweight	Yes	Yes	Yes	No
E: Stunting and underweight	No	Yes	Yes	No
F: Stunting only	No	Yes	No	No
Y: Underweight only	No	No	Yes	No
G: Stunting and Overweight	No	Yes	No	Yes
H: Overweight alone	No	No	No	Yes

**FIGURE 2:**
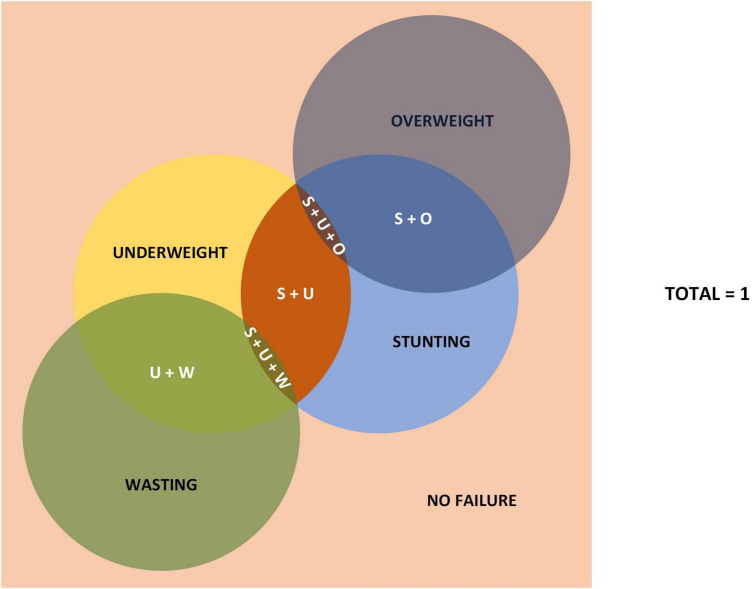
Newly proposed model to measure all forms of nutritional status. Not to scale. U + W, underweight and wasting; S + O, stunting and overweight; S + U, stunting and underweight; S + U + O, stunting, underweight, and overweight; S + U + W, stunting, underweight, and wasting.

Keeping the total sample size used for analysis same between the two models, the revised model by Kuiti and Bose et al. (5), should show no change in the category of “Stunting and Underweight” when compared to Svedberg’s model. However, when we did the NFHS-5 data analysis with both the models, we found a decrease in the number of samples in “Stunting and Underweight” category in Kuiti’s model compared to Svedberg’s model. This discrepancy between the two models led us to hypothesize that there must be an additional one or more categories. We identified the category called “Stunting, Underweight, and Overweight.” When we did the data analysis after adding this new variable, the number of samples under the new category of “Stunting, Underweight and Overweight” were same as the number of samples decreased from the “Stunting and Underweight” category in Kuiti’s model. This was also the same number seen as additional samples in the “Stunting and Overweight” category in Kuiti’s model (Figure 2). The various categories of nutritional status in the new model are shown in Table 3.

**TABLE 3:** All the groups in the new proposed model.

Group	Wasting	Stunting	Underweight	Overweight
A: No failure	No	No	No	No
B: Wasting only	Yes	No	No	No
C: Wasting and underweight	Yes	No	Yes	No
D: Stunting, wasting and underweight	Yes	Yes	Yes	No
E: Stunting and underweight	No	Yes	Yes	No
F: Stunting only	No	Yes	No	No
Y: Underweight only	No	No	Yes	No
G: Stunting and Overweight	No	Yes	No	Yes
H: Overweight only	No	No	No	Yes
I: Stunting, underweight, and overweight	No	Yes	Yes	Yes

Theoretically, there is also the possibility of underweight and overweight occurring together without stunting. But it was not seen from the NFHS-5 data. Therefore, we hypothesize that underweight and overweight can co-exist in the same child only with stunting, and it is biologically implausible to have underweight and overweight together without stunting.

However, there are issues with looking at HAZ, WAZ, and WHZ separately. Human growth in length or height occurs in aperiodic saltatory spurts that are discontinuous and interspersed by periods of no measurable growth (6, 7). Cross-sectional surveys like NFHS-5 cannot take this into account and might misclassify a growing child as malnourished. For example, when a child is taller for his age, the measurement of weight-for-height tends to overestimate wasting. When HAZ is > +1SD and WAZ is > −2SD, which may occur during growth process, the child is within normal limits and age-appropriate. But the corresponding WHZ might be less than −2SD and the child may be categorized as wasted. An individual child may suffer from both stunting and wasting, called concurrence (8). Nevertheless, they are considered separately, especially with respect to how they are managed programmatically and clinically (9, 10).

Creating a new score that incorporates the three anthropometric Z-scores (HAZ, WAZ, and WHZ) can help solve the problem. This score should, to some extent, adjust for the fluctuations in the differences in rate of linear and ponderal growth in under five children as it considers the various forms of anthropometric failure together for the calculation of the score. With this background, our objective is to develop a new comprehensive scoring system incorporating HAZ, WAZ, and WHZ, and using the new scoring system, demonstrate the different forms and combinations of malnutrition among under five children in the different groups of the proposed model, using the National Family Health Survey-5 (NFHS-5) data from India.

## Materials and methods

### Computation of the score

A composite score is more comprehensive as it includes more than one dimension of the growth and development of the child and thus reflects more than one aspect of nutrition. Talwar (11) attempted to develop an index of nutritional level from anthropometric measurements in pre-school children using height and weight as a percentage of the standards. It was suggested to have an average of the indicators, which could also serve as an index of nutrition state of the child (12). But, taking an average of indicators will not work with Z-scores as they include both positive as well as negative values, and they can cancel each other out. This could result in wrongly classifying a malnourished child as having no anthropometric failure.

To solve this, we propose subtracting the Z-scores from a value which will be variable “a.” e.g., the structure of the score would be: a−(HAZ + WAZ + WHZ).

But WHZ > +2SD as well as WHZ < −2SD are both considered anthropometric failures and must be accounted for in the formulas. Therefore, if WHZ < 0, then the score should take up the form of:

[a-(HAZ+WAZ+WHZ)]

While, if WHZ > 0, the score should be:

[a-(HAZ+WAZ-WHZ)]

Since HAZ ranges from −6 to +6, WAZ ranges from −6 to +5, and WHZ ranges from −5 to +5, we can see that the maximum possible value either HAZ + WAZ + WHZ (when WHZ < 0) or HAZ + WAZ−WHZ (when WHZ > 0) can take up is when (HAZ + WAZ) is maximum and WHZ = 0. This happens when HAZ = +6 and WAZ = +5.

Therefore, the maximum possible value that either (HAZ + WAZ + WHZ) or (HAZ + WAZ−WHZ) can take is 6 + 5 = 11.

Therefore, to keep the minimum value of the score to 0, we can assign the value of the variable “a” as 11.

Hence, the formula becomes:

11-(HAZ+WAZ+WHZ),when⁢WHZ<0

11-(HAZ+WAZ-WHZ),when⁢WHZ>0

We can see that the maximum possible value of either score is 28 and that occurs when HAZ = −6, WAZ = −6, and WHZ = −5 (i.e., when each of the parameter takes their lowest possible value).

To keep the range of the score from 0 to 100, we can convert formulas as:

$$\frac{100}{28}\,x\,\left(11-\left(\mathrm{HAZ}+\mathrm{WAZ}+\mathrm{WHZ}\right)\right),\mathrm{when}\,\mathrm{WHZ}<0$$

$$\frac{100}{28}\,x\,\left(11-\left(\mathrm{HAZ}+\mathrm{WAZ}-\mathrm{WHZ}\right)\right),\mathrm{when}\,\mathrm{WHZ}>0$$

We found that, using these formulas, although the canceling between positive and negative Z-scores decreased, they still cancel each other out significantly. To solve this issue, and to give more weightage to the Z-scores that are at the extreme ends of anthropometric failure (to give more weightage to severe failure), we found a solution in raising the HAZ, WAZ and WHZ scores to various powers of integers.

If “n” is the power to which the scores are to be raised, the equation simplifies as:

$$\text{Score}=\frac{100}{\left(3\times 6^{n}\right)+\left(2\times 5^{n}\right)}\times [\left(6^{n}+5^{n}\right)-((HAZ^{2\,n-1}\text{÷}$$

$$|HAZ^{n-1}|)+(WAZ^{2\,n-1}\text{÷}|WAZ^{n-1}|)+(WHZ^{2\,n-1}\text{÷}$$

$$\left|WHZ^{n-1}|\right))],\mathrm{when}\mathrm{WHZ}<0$$

 

$$\text{Score}=\frac{100}{\left(3\times 6^{n}\right)+\left(2\times 5^{n}\right)}\times [\left(6^{n}+5^{n}\right)-((HAZ^{2\,n-1}\text{÷}$$

$$|HAZ^{n-1}|)+(WAZ^{2\,n-1}\text{÷}|WAZ^{n-1}|)-(WHZ^{2\,n-1}\text{÷}$$

$$\left|WHZ^{n-1}|\right))],\mathrm{when}\mathrm{WHZ}>0$$

We have used, for example, (*HAZ*^2*n*−1^÷|*HAZ*^*n*−1^|) instead of just *HAZ^n^* because *HAZ^n^* might convert the negative values to positive if ‘n’ is an even number.

A higher score implies more severe malnutrition. The value of “n” can be chosen for the best sensitivity and specificity. In this paper, we have taken the value of “n” as 15 (to get a specificity of 100%). We then arranged the children in the ascending order of the new score. The cut-off is taken as the value that corresponds to the lowest score that contains HAZ < (−2.10), which is 34.02459913666280 in this case. Scores more than this cut-off are considered “Failure,” while scores less than this cut-off are considered “No-failure.” The new scoring system has a specificity of 100% (no child without any form of anthropometric failure is considered under the “Failure” category using the new score) and a sensitivity of 93.25% (93.25% children considered “No Failure” using the new score does not have any form of anthropometric failure, while 6.75% of children who were previously considered to have anthropometric failure is also included in this category) (Table 4).

**TABLE 4:** Sensitivity and specificity of the new scoring system (Unweighted).

		Considered as failure if HAZ, WAZ, or WHZ < −2; or WHZ > +2		
		**Failure**	**No failure**	**Total**
New scoring system	Failure	97,690	0	97,690
	No failure	7,733	93,379	101,112
	Total	105,423	93,379	198,802

### Data used for demonstrating the application of developed score

For demonstrating the application of this score, we used data from the fifth round of the National Family Health Survey (NFHS-5, 2019-21). The NFHS-5 is a household survey conducted in 2019-21, covering all the States and Union Territories of India. This survey is designed to generate estimates of population, health, and nutrition at national, state/union territory (UT), and district levels. Four survey schedules (Household, Woman’s, Man’s, and Biomarker) were canvassed in 636,699 households across India using Computer Assisted Personal Interviewing (CAPI). The NFHS-5 has collected information of 724,115 women (15–49 years), 101,839 men (15–54 years), and 232,920 children (0–59 months). The height and weight of children (0–59 months) were measured and recorded in the NFHS-5. The weight of children (0–59 months) was measured using the Seca 874 digital scale. The height of children (24–59 months) was measured with the Seca 213 stadiometer. The Seca 417 Infantometer was used to measure the recumbent length of children under two years or less than 85 cm. The WHO Z-scores for HAZ (height-for-age), WHZ (weight-for-height), and WAZ (weight-for-age) were also available in the data set.

### Statistical analysis

For this analysis, we used the NFHS-5 data set (IAKR7AFL file) in Stata format downloaded from the Demographic Health Survey (DHS) program portal. Individual level data of 232,920 children between the ages of 0 and 59 months was available in the dataset. By using HAZ, WHZ, and WAZ variables, we have derived nine biologically possible categories of malnutrition, according to the new model, among children under five years of age.

1. Children with only stunting [HAZ < −2SD]
2. Children with only wasting [WHZ < −2SD]
3. Children with only underweight [WAZ < −2SD]
4. Children with stunting, wasting and underweight [HAZ < −2SD & WHZ < −2SD & WAZ < −2SD]
5. Children with stunting and underweight [HAZ < −2SD & WAZ < −2SD]
6. Children with wasting and underweight [WHZ < −2SD & WAZ < −2SD]
7. Children with stunting and overweight [HAZ < −2SD & WHZ > +2SD]
8. Children with only overweight [WHZ > +2SD]
9. Children with stunting, underweight and overweight [HAZ < −2SD & WAZ < −2SD & WHZ > +2SD]

In this analysis, we estimated the proportion of different forms of malnutrition among children (0–59 months) along with 95% confidence interval. All the analysis has been carried out using the Stata-14 statistical software package developed by StataCorp.

## Results

**FIGURE 3:**
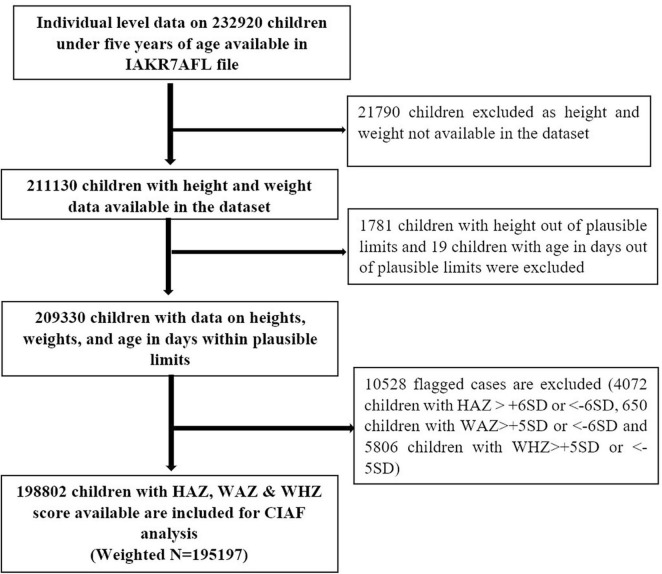
Flow diagram depicting the inclusion and exclusion criteria followed for the final analysis using NFHS-5 data. CIAF, Composite Index of Anthropometric Failure; HAZ, height-for-age Z-score; NFHS, National Family Health Survey; SD, standard deviation; WAZ, weight-for-age Z-score; WHZ, weight-for-height Z-score.

There was individual level data on 232,920 under five children in NFHS-5 data file. We excluded 21,790 children from the data set as height and weight data were not available. Another 1,781 children were excluded as they had a height out of plausible limits, and 19 children were of age in days out of plausible limits. Another 10,528 children were excluded from the data set as they were flagged with Z-score beyond plausible limits (4,072 children with HAZ > +6SD or < −6SD, 650 children with WAZ > +5SD or < −6SD, and 5,806 children with WHZ > +5SD or < −5SD). Our final analysis included individual level data of 198,802 children under five years of age (weighted *N* = 195,197) (Figure 3).

Using the new scoring system, stunting, wasting, underweight, and overweight decreased by 1, 1.6, 2.2, and 0.5% from the values given in the NFHS-5 data (Table 5). After applying the new scoring system to the proposed model, the prevalence of stunting has reduced to 11.8% (95% CI: 11.66–11.94) from 13.2% (95% CI: 13.09–13.39). Similarly wasting prevalence has reduced to 4.9% (95% CI 4.85–5.04) after applying the new scoring system to proposed model from 6.4% (95% CI: 6.29–6.51). As per our analysis, the most common forms of anthropometric failures among Indian children are: “Stunting and underweight” (30,131; 15.4%), Stunting only (25,851; 13.2%), and “Wasting and underweight” (14,698; 7.5%). We found a new category called “Stunting, underweight, and overweight” (stunting = HAZ < −2SD, underweight = WAZ < −2SD, overweight = WHZ > +2SD). It constituted 0.1% (220 children) of the total sample of children (0–59 months) (Table 6). In this category, HAZ score ranges from −3.82 to −6.00, WAZ score ranges from −2.01 to −4.03, and WHZ score ranges from +2.01 to +4.82.

**TABLE 5:** Proportion of under-five children in India having stunting, wasting, underweight, and overweight using the proposed model after applying the scoring system compared to the NFHS-5 data [weighted frequency = 195,197].

Category	Weighted frequency using the proposed model after applying the scoring system	Weighted proportion (%) using the proposed model after applying the scoring system	Proportion (%) provided in NFHS-5 survey (33)
Stunting	67,254	34.5	35.5
Wasting	34,568	17.7	19.3
Underweight	58,385	29.9	32.1
Overweight	5,601	2.9	3.4

**TABLE 6:** Proportion of under-five children in India as per their nutritional status using the proposed models from NFHS-5 data [Weighted frequency = 195,197].

Sl no.	Category	Proposed model		Proposed model after applying the new score	
		**Weighted frequency**	**Weighted proportion (%) [95% CI]**	**Weighted frequency**	**Weighted proportion (%) [95% CI]**
1	Stunting only [HAZ < −2SD]	25,851	13.2 [13.09–13.39]	23,035	11.8 [11.66–11.94]
2	Wasting only [WHZ < −2SD]	12,494	6.4 [6.29–06.51]	9,656	4.9 [4.85–5.04]
3	Underweight only [WAZ < −2SD]	4,467	2.3 [02.22–02.36]	3,125	1.6 [1.55–1.66]
4	Overweight only [WHZ > +2SD]	2,189	1.1 [01.08–01.17]	1,724	0.9 [0.84–0.93]
5	Stunting and underweight [HAZ < −2SD & WAZ < −2SD]	30,131	15.4 [15.28–15.60]	30,127	15.4 [15.27–15.60]
6	Wasting and underweight [WHZ < −2SD & WAZ < −2SD]	14,698	7.5 [07.41–07.65]	14,698	7.5 [7.41–7.65]
7	Stunting and overweight [HAZ < −2SD & WHZ > +2SD]	3,657	1.9 [01.81–01.93]	3,657	1.9 [1.81–1.93]
8	Stunting, wasting and underweight [HAZ < −2SD & WHZ < −2SD & WAZ < −2SD]	10,214	5.2 [05.13–05.33]	10,214	5.2 [5.13–5.33]
9	Stunting, underweight and overweight [HAZ < −2SD & WAZ < −2SD & WHZ > +2SD]	220	0.1 [0.10–0.13]	220	0.1 [0.10–0.13]
10	No failure	91,276	46.8 [46.54–46.98	98,740	50.6 [50.36–50.81]

## Discussion

We propose a template of a new score that combines the three Z-scores to provide a holistic approach to malnutrition in a child. We also propose a model to describe all the forms of nutritional status among children under five years of age into discrete atomistic groups without any overlap among the categories. On applying the new score to the proposed model, we found that 15.4% of children were “stunting and underweight” while 11.8% of the children were “only stunting,” and they constituted the groups with the largest burden of malnutrition. This method of a holistic approach followed by dividing them into discrete categories prevents wrongly classifying a normally growing child as malnourished as well as captures all forms and combinations of anthropometric failure among children under five years of age instead of the current overlapping method of demonstrating malnutrition.

A new category of malnutrition called “Stunting, underweight, and overweight” was described for the first time (to our knowledge). We have demonstrated using the NFHS-5 data that the possibility of underweight (WAZ < −2SD) and overweight (WHZ > +2SD) occurring together in the same individual is also seen practically in the community in a small proportion of stunted children.

Our model can identify the most severe combination of anthropometric failure, namely “Stunting, wasting, and underweight.” In a meta-analysis of individual data in 10 prospective studies from developing countries to study the effect of multiple anthropometric failures on child mortality, it was found that the risk of mortality increased with more deficits (13). Having stunting, wasting and underweight together was associated with the greatest risk of mortality and was twelve times that of children with no anthropometric failure (13). Further research needs to be done to identify the risk of child mortality and morbidity in the newly identified category of “Stunting, Underweight, and Overweight.” Since the new scoring system considers HAZ, WAZ and WHZ scores together, it can be used to identify the children having the highest risks.

We are not certain if the normal methods of rehabilitation might be helpful in this small proportion of children who are underweight, overweight, and stunting. Without the improvement in stunting, they either remain underweight or feeding them more might cause the overweight component to worsen. But stunting is often irreversible (8, 14), although periods of “catch-up” growth have been described (15). Therefore, we must be very judicious in treating the children belonging to this group.

Most studies use the initial CIAF model that divides malnutrition into six-categories (and an additional category of no anthropometric failure) (16–19). But this fails to consider overweight and thereby underestimates the total burden of malnutrition. We suggest renaming Overweight (> +2SD weight-for-height) to “Overnourished” as the former term is often mistakenly considered to be the opposite of Underweight (< −2SD weight-for-age).

The limitations of our paper are that, in calculating the score, we have also included WAZ score (Underweight = WAZ < −2). But “underweight” was introduced as a single indicator to incorporate both stunting and wasting (3). It is not ideal to use WAZ score for the computation of the score. However, we have used WAZ to categorize into the 9 groups of anthropometric failure which include underweight as well. Another drawback of the score is that the range of HAZ, WAZ, and WHZ are different (HAZ score ranges from +6 to −6, WAZ score ranges from +5 to −6, and WHZ score ranges from +5 to −5). This can affect the score at the extreme values. Ideally, while doing the analysis, the ranges of all the Z-scores should be kept the same. However, since these are pre-determined ranges, we decided not to change them. Therefore, we recommend using this score only as a template for further development of a comprehensive score of malnutrition. It is also worth mentioning that this score should not be used for identifying children with severe acute malnutrition (SAM) or moderate acute malnutrition (MAM), as some children with SAM or MAM might not be considered as having anthropometric failure using the new score if their HAZ scores are high.

We have taken the arbitrary cut-off point for the new scoring system as the value that corresponds to the lowest score that contains HAZ < −2.10, and not −2 even though this resulted in a lesser sensitivity of the score. However, this will help in the identification of a greater number of children who might be misclassified as malnourished. In the Reviews Analysis, World Health Organization (WHO) Working Group (2) states that the use of fixed cut-off points such as −2SD or its equivalent may be unrealistic and of limited use in practice (20–22), and that at best, they represent a purely statistical separation of “malnourished” from “normal” when ideally cut-off points should be based on biological considerations. We also kept this in mind when we chose the cut-off for the new score mentioned above. HAZ < −2 does not consider linear growth faltering. The anthropometric indicators are only proxies for the physiological and functional effects of undernutrition (23). The use and interpretation of indicators such as HAZ < −2SD has uncoupled from its actual use as a population level indicator (24). This is in contrast to the guidelines provided by WHO on the use and interpretation of anthropometry (25). Reliance on anthropometry leads to a false division and categorization of childhood undernutrition. Knowing this, we have still based our models on anthropometric indicators due to its global applicability, ease of use, low cost, and non-invasiveness (2). However, we would like to study ways of determining childhood malnutrition that have biological significance and is less reliant on the current anthropometric indicators.

The strength of our model is that it captures all forms of nutritional status among children under five years of age. This helps us to identify the discrete forms as well as various combinations and gives the exact burden of each kind of anthropometric failure without any overlap. The score would be useful in identifying the children in communities with severe growth failure of various presentations. It would also be helpful for targeted programmatic intervention by preventing missing-out of any child that slipped through the severity category for stunting/wasting/overweight/underweight but had a composite growth failure.

Studies done in the recent years have used CIAF to assess the nutritional status or of undernutrition, but have not taken into account the category of stunting, overweight, and underweight (26–29). CIAF has also been used to find the determinants of malnutrition at various age groups (30–32). We could not find any study that has tried to further modify the CIAF beyond the works that are already mentioned in this manuscript. Further studies need to be done to identify the predictors of the new group of malnutrition involving three indicators namely “stunting, underweight, and overweight” and their effects on children later in life. Since the majority of anthropometric failures occur in combinations, policies that focus on only one form at a time may not yield the expected results.

In conclusion, we have developed a new scoring system that considers various forms of malnutrition (based on Z-scores) instead of a single anthropometric indicator to classify a child as malnourished and a proposed model of CIAF to capture all forms of nutritional status among under five children. We then used this scoring system on the proposed model. The advantages of describing anthropometric failure in this way are that it prevents misclassifying a growing child as malnourished and individual forms of malnutrition can be captured without any overlap, revealing us the groups of malnutrition with the greatest burden. Major nutritional surveys can use this model for description as it helps researchers and policy makers for right strategies. The new category identified in our model shows that stunting (HAZ < −2SD), overweight (WHZ > +2SD), and underweight (WAZ < −2SD) can co-exist in the same child.

## Data availability statement

Publicly available datasets were analyzed in this study. This data can be found here: https://dhsprogram.com/methodology/survey/survey-display-541.cfm.

## Author contributions

EN: Data curation, Formal analysis, Investigation, Methodology, Software, Visualization, Writing – original draft. AJ: Data curation, Formal analysis, Methodology, Visualization, Writing – original draft. PR: Writing – original draft. JG: Project administration, Supervision, Writing – review & editing. SR: Conceptualization, Project administration, Supervision, Validation, Visualization, Writing – review & editing. RH: Conceptualization, Funding acquisition, Resources, Validation, Visualization, Writing – review & editing.
